# Specific cognitive functions and depressive symptoms as predictors of activities of daily living in older adults with heterogeneous cognitive backgrounds

**DOI:** 10.3389/fnagi.2015.00139

**Published:** 2015-07-20

**Authors:** Jonas J. de Paula, Breno S. Diniz, Maria A. Bicalho, Maicon Rodrigues Albuquerque, Rodrigo Nicolato, Edgar N. de Moraes, Marco A. Romano-Silva, Leandro F. Malloy-Diniz

**Affiliations:** ^1^Faculdade de Medicina, Instituto Nacional de Ciências e Tecnologia e em Medicina Molecular, Universidade Federal de Minas GeraisBelo Horizonte, Brazil; ^2^Department of Psychology, Faculdade de Ciências Médicas de Minas GeraisBelo Horizonte, Brazil; ^3^Department of Mental Health, Faculdade de Medicina, Universidade Federal de Minas GeraisBelo Horizonte, Brazil; ^4^Department of Internal Medicine, Faculdade de Medicina, Universidade Federal de Minas GeraisBelo Horizonte, Brazil; ^5^Department of Physical Education, Universidade Federal de ViçosaViçosa, Brazil

**Keywords:** activities of daily living, functional performance, neuropsychological assessment, depression, dementia, mild cognitive impairment, executive functions

## Abstract

Cognitive functioning influences activities of daily living (ADL). However, studies reporting the association between ADL and neuropsychological performance show inconsistent results regarding what specific cognitive domains are related to each specific functional domains. Additionally, whether depressive symptoms are associated with a worse functional performance in older adults is still under explored. We investigated if specific cognitive domains and depressive symptoms would affect different aspects of ADL. Participants were 274 older adults (96 normal aging participants, 85 patients with mild cognitive impairment, and 93 patients probable with mild Alzheimer’s disease dementia) with low formal education (∼4 years). Measures of ADL included three complexity levels: Self-care, Instrumental-Domestic, and Instrumental-Complex. The specific cognitive functions were evaluated through a factorial strategy resulting in four cognitive domains: Executive Functions, Language/Semantic Memory, Episodic Memory, and Visuospatial Abilities. The Geriatric Depression Scale measured depressive symptoms. Multiple linear regression analysis showed executive functions and episodic memory as significant predictors of Instrumental-Domestic ADL, and executive functions, episodic memory and language/semantic memory as predictors of Instrumental-Complex ADL (22 and 28% of explained variance, respectively). Ordinal regression analysis showed the influence of specific cognitive functions and depressive symptoms on each one of the instrumental ADL. We observed a heterogeneous pattern of association with explained variance ranging from 22 to 38%. Different instrumental ADL had specific cognitive predictors and depressive symptoms were predictive of ADL involving social contact. Our results suggest a specific pattern of influence depending on the specific instrumental daily living activity.

## Introduction

Cognitive and functional impairments are hallmarks of cognitive disorders and defining features of mild cognitive impairment (MCI) and dementia. In MCI, the cognitive deficits do not impair the capacity to live independently, in contrast to individuals with dementia that present pronounced functional deficits, such as the ones observed in Alzheimer’s disease (AD; (Pereira et al., 2010; Brown et al., 2011; Seelye et al., 2013). The most usual form to assess functional performance in older adults is the investigation of activities of daily living (ADL), common activities performed by the majority of older adults in a specific cultural setting (Lawton and Brody, 1969).

Prior studies have investigated the relationship between cognitive and functional performance in older adults with MCI or AD. Longitudinal changes in cognition are related to longitudinal changes in ADL (Farias et al., 2009). In a comprehensive review, Royall et al. (2007) showed a weak to moderate association between global cognitive measures and functional impairment. However, their results are heterogeneous with cognitive features responding for 0 to 80% of the variance in functional performance (mean of 21% with a SD of 20%; Gold, 2012). Methodological differences and sample characteristics might explain part of this excessive variability.

Gold (2012) discuss some of the methodological issues. The definition and the type of ADL investigated varies between studies. Some studies focuses on a unitary construct of ADL, in contrast with several evidences from the literature of a multidimensional construct involving activities of different levels of complexity (Thomas et al., 1998; Niti et al., 2007; Gold, 2012; de Paula et al., 2014). Beyond the usual distinction between Basic (BADL) and Instrumental (IADL) activities, some studies found different latent structures in ADL questionnaires and scales. Thomas et al. (1998) reported a multidimensional structure for BADL and IADL combined, proposing its interpretation based on levels of complexity (basic, intermediate, and complex). Niti et al. (2007) found a bifactorial structure for IADL (“physical” and “cognitive”) in a sample of Asian older adults with a significant influence of specific cultural aspects on participants’ responses. In a sample of low educated older adults from Brazil, de Paula et al. (2014) observed a different factorial solution with a “Domestic” IADL component and a “Complex” IADL component, in addition to a third component classified as BADL. Therefore, ADL may be a multidimensional construct with different components varying according to sample characteristics (Bootsma-van der Wiel et al., 2001; Cabrero-García and López-Pina, 2008; Fieo et al., 2011). Such differences might account for some of the high heterogeneity between studies reported in Royall et al. (2007). This emphasizes the importance of investigating specificities in ADL structure (Gold, 2012).

Methodological difficulties include instrument bias. Sikkes et al. (2009) reviewed the literature concerning the different measures of IADL focusing on its psychometric properties. Their results suggest that most of the scales adopted in clinical and research settings still lack of psychometric studies, reducing its validity and reliability for the functional assessment, although great effort is being dispend on this matter (Fieo et al., 2011). The source of information (e.g., patient vs. caregiver) is also not consensual in studies including cases of dementia, which is of extreme importance to the use and interpretation of scales measurement (Farias et al., 2005). Bootsma-van der Wiel et al. (2001) also highlight the conceptual difference between a “can do” or an “actually do” score in specific activities. Additionally, there is no consensus in the literature indicating scales and questionnaires as adequate methods to functional level measurement. There are more ecological measures of ADL, which involve the observation of the patient’s behavior on real life or simulated settings (a more precise estimation of functional performance; Chaytor and Schmitter-Edgecombe, 2003). However, these procedures also have limitations such as the higher costs of execution and the inherent complexity of the assessment, and might not be well suited for most of the clinical and research settings. The suitability of these ecological measures for studies involving cognitive performance is controversial. There are studies showing a stronger association of cognitive measures with ecological measures of ADL (Burton et al., 2006; Tam et al., 2008). However, the opposite pattern was also demonstrated with the relation between cognitive performance with ADL being stronger for ADL measured by scales/questionnaires (Cahn-Weiner et al., 2002; Mariani et al., 2008; de Paula et al., 2014). The cognitive processes assessed by the neuropsychological tests used in these studies might be associated with ADL in different and more specific ways (Gold, 2012).

Taking specific cognitive domains as predictors of functional performance most of the studies report associations with executive functions (Royall et al., 2007; Pereira et al., 2008; de Paula and Malloy-Diniz, 2013). This complex cognitive construct usually shows the strongest correlations with functional performance. Executive functions involve planning, initiation, monitoring, inhibition, and flexibility of goal-oriented behavior (Diamond, 2013). These specific aspects of executive functions may contribute differently to the ADL (Jefferson et al., 2006). However, other cognitive functions may contribute to specific aspects of ADL. Cognitive measures of spatial processing predicted participants’ performance in an ecological measure of visuospatial abilities in which the subject had to estimate distances, positions, and directions in a “real-life” setting developed by Farley et al. (2011). Activities demanding communicative skills were related to semantic process and language (Razani et al., 2011). Schmitter-Edgecombe et al. (2009) investigated different aspects of episodic memory and its association with ADL reporting significant associations of specific memory components with specific functional components. In this sense, although most of the studies have focused on executive functions or global cognitive measures, different kinds of ADL may depend on different cognitive abilities.

Depressive symptoms can also impair functional performance in older adults, usually in more complex activities (Bombin et al., 2012; Tomita and Burns, 2013; Zahodne et al., 2013; Park et al., 2014). Our group reported a weak association between depressive symptoms and functional performance in older adults with low formal education diagnosed with MCI or AD (de Paula and Malloy-Diniz, 2013). A stronger association was recently reported in a similar sample (Assis et al., 2014). Cahn-Weiner et al. (2002) found an association of depressive symptoms with ADL independent from cognitive functioning. Remission of depressive symptoms is associated with improvement of ADL (Nyunt et al., 2012). Nonetheless, there is evidence for the contrary, suggesting that depressive symptoms are not associated with IADL after controlling for cognitive symptoms (Reppermund et al., 2011; Wadsworth et al., 2012). Then, whether depressive symptoms affect functional performance by behavioral symptoms (depressed mood, lack of pleasure, apathy, and vegetative symptoms) or through cognitive impairment associated with depression remains unclear.

Functional deficit is one of the hallmarks of MCI and AD in older adults. Improving cognitive functions and mood symptoms may result in gains in functional performance. Therefore, a better understanding of how specific cognitive abilities and depressive symptoms contribute to the performance of specific ADL could be important to the development of tailored rehabilitation programs to improve daily functioning in individuals with cognitive disorders in a personalized way. The objective of the present study is to assess how specific cognitive abilities and symptoms of depression are associated to different aspects of ADL.

## Materials and Methods

### Participants

We evaluated 274 older adults from a public outpatient clinic specialized in cognitive disorders and frailty. The center usually receives elderly patients referred from primary-care physicians when they suspect of cognitive impairment, mental disorders, or multiple chronic diseases. Patients usually have a very low socioeconomic status and less than 4 years of formal education. Participants’ sociodemographic characteristics are shown in **Table 1**. A more detailed description of the typical profile of patients assessed in this center was published elsewhere (Bicalho et al., 2013; de Paula et al., 2013a).

**Table 1:** Participants’ demographic profile.

Cognitive status	Normal aging	35%
	Mild cognitive impairment	31%
	Alzheimer’s disease dementia	34%

Gender	Male	39%
	Female	61%

Depression^1^	Present	30%
	Absent	70%

Age	60–69 years	34%
	70–79 years	43%
	80+ years	23%

Formal education	Illiterate	12%
	1–4 years	57%
	5–8 years	13%
	9+ years	12%
	12+ years	6%

Occupations^2^	Craft and related trades workers	13%
	Elementary occupations	34%
	Service and sale workers	22%
	Others	31%

Retired?	No	13%
	Yes	87%

Marital status	Married	53%
	Divorced	12%
	Single	9%
	Widow	36%

*^1^According to the Geriatric Depression Scale 15 cut-off (5/6).*

*^2^According to the International Labour Office (ILO, 2012).*

The participants underwent a detailed clinical, cognitive, and behavioral assessment for diagnostic purposes as described below. During the geriatrician examination and the clinical neuropsychological assessment, the patients underwent cognitive, functional and behavioral assessment to determine their cognitive status. Ninety-three participants were diagnosed with mild Alzheimer’s disease dementia (AD), 85 patients were diagnosed with amnestic MCI (MCI) and 96 older adults were normal aging participants without clinical history, cognitive, or functional status suggestive of dementia or AD. AD was diagnosed by the NINCDS-ADRDA criteria for probable dementia (McKhann et al., 1984). Only patients with mild dementia, according to Clinical Dementia Rating (CDR; Morris, 1993), were invited for participation in this study. MCI diagnosis was based on a modified version of the Mayo Clinic diagnostic criteria (Petersen et al., 2001). Criteria for MCI was as follow:

(1) Subjective cognitive complaint, preferably corroborated by an informant/caregiver.
(2) Objective impairment on specific cognitive measures of the assessment battery for diagnosis according to Brazilian norms and cut-off scores (Porto et al., 2003; Nitrini et al., 2004). The cognitive battery includes the Verbal Learning Test of the CERAD Neuropsychological Battery (Morris et al., 1989), the memory test from the Brief Cognitive Battery (Nitrini et al., 2007), and subscales of the Mattis Dementia Rating Scale (Mattis, 1988).
(3) Normal global cognitive functioning (MMSE above the cut-off for dementia and CDR < 1).
(4) Preserved or minimal impairments in ADL assessed by a clinical interview and the CDR.
(5) Not demented based on the DSM-IV-TR criteria (American Psychiatric Association [APA], 2000).

All groups (i.e., AD, MCI, and control) were combined in a unique heterogeneous sample. This strategy was adopted to increase statistical power and to avoid detection loss of cognitive processes that are likely to underlie functional performance (Farias et al., 2009; Gold, 2012).

### Cognitive, Functional, and Mood Assessment

We adopted an unstructured protocol of neuropsychological tests designed for the assessment of older adults with low formal education. The tests were not included in patients’ diagnosis. Cognitive composite factors were obtained through factor analysis from our sample (statistical procedures detailed below). This approach allows the assessment of different aspects of cognitive functioning (here, the core domains recommended for the MCI and AD diagnosis) with greater specificity. The protocol comprised tests of executive functions (Frontal Assessment Battery, Verbal Fluency tests, and Digit Span); language and semantic memory (Laboratory of Neuropsychological Investigations Naming Test – Nouns, Verbs and Professions, Token Test verbal comprehension component); episodic memory (components of learning, recognition, immediate, and delayed recall of the Rey Auditory-Verbal Learning Test); and visuospatial abilities (Clock Drawing Test, Stick Design Test, and Token Test visual attention components). These tests are valid and reliable for the assessment of older adults with a low educational background (de Paula et al., 2013a). The test measures are shown in **Table 2**.

**Table 2:** Neuropsychological measures used to extract the four cognitive factors according to de Paula et al. (2013a).

Cognitive domain	Test	Test measures	Reference
Executive functions	Frontal assessment battery	Total score	Dubois et al. (2000)
	Verbal fluency	Animals	Lezak et al. (2004)
		Fruits	
		Letter “S”	
	Digit span forward	Correct trials × Span	Kessels et al. (2008)
	Digit span backward	Correct Trials × Span	Kessels et al. (2008)
Language semantic memory	TN-LIN (naming test)	Nouns	Malloy-Diniz et al. (2007a)
		Actions	
		Professions	
Episodic memory	RAVLT	Short term memory (A1)	Malloy-Diniz et al. (2007b)
		Immediate recall (A6)	
		Delayed recall (A7)	
		Sum of words	
		Recognition memory	
Visuospatial abilities	Stick design test	Total score	Baiyewu et al. (2004)
	Clock drawing test	Total score	Shulman (2000)
	Token test (short version)	Visual attention	De Renzi and Faglioni (1978)
		Complex comprehension	

*TN-LIN, Naming test of the laboratory of neuropsychological investigations; RAVLT, Rey auditory-verbal learning test.*

The assessment of ADL occurred during the clinical and neuropsychological assessment by an interview with participants’ caregivers. For this study, we used the ‘General Activities of Daily Living Scale’ (GADL) (de Paula et al., 2014), a multidimensional functional measure of BADL/IADL based on the Lawton and Brody (1969) and Katz et al. (1970) indexes of ADL. The GADL shows a hierarchical structure with a general score and three components of more specific activities: a measure of BADL (Self-care: ability to change clothes, use the toilet, use the shower, transference from bed or chair, and feed itself) and two components of IADL. Instrumental Domestic ADL include ability to perform domestic chores, use the telephone, prepare meals, and do the laundry. Instrumental Complex ADL include ability to manage financial matters, shopping, adequate use of medication, and go out alone using transportation. This structure was determined by factor analysis on the same sample of the present study and showed evidence of reliability and validity (de Paula et al., 2014). We scored each activity using a 3-point Lickert scale (dependent, partially dependent, or independent of assistance to perform the activity). The GADL subscores of Self-care (0–10), Instrumental-Domestic (0–8), Instrumental-Complex (0–8), and the Global score (0–26) represented the general ADL measures in our study. To investigate the association of different cognitive functions with specific measures of functional performance, we also used each item of the scale (13 different ADL) independently.

We assessed the depressive symptoms with the Brazilian version of the Geriatric Depression Scale-15 (GDS-15; Sheikh and Yeasavage, 1986). A validation study conducted in Brazil attested its sensitivity and specificity for the detection of depression (Almeida and Almeida, 1999). However, since our focus was not to identify patients with major depressive disorder, but to use a dimensional measure of its symptoms, we used the GDS-15 total score in this research. The GDS use for depression diagnosis in dementia is controversial. To reduce biases we selected only patients with mild dementia for the AD group (CDR ≤ 1). Due to participants’ low formal education, the examiner read the GDS questions aloud to ensure the comprehension and validity of patients’ report.

### Statistical Procedures

Our four cognitive domains were extracted by factor analysis (principal axis factoring with an oblique rotation of the neuropsychological tests described in Cognitive Assessment) from our sample. The procedures were described in detail elsewhere (de Paula et al., 2013a). Briefly, the cognitive factors were saved by a regression method and standardized (*Z*-Score) based on the performance of our cognitively normal non-depressed participants. The four factors were *executive functions, episodic memory, language/semantic memory*, and *visuospatial Abilities*. These factors showed high internal consistency and reliability (Cronbach’s alpha > 0.800 for all factors).

We carried out univariate analysis of variance (continuous variables) or chi-square tests (categorical variables) to evaluate baseline differences in sociodemographic, clinical, cognitive, and ADL measures between the AD, MCI, and control groups. Part of this data was previously published (de Paula et al., 2013a). For a preliminary assessment of the relationship between cognitive functioning, depressive level and ADL, we correlated each measure with the GADL global score. The influence of age, education, and gender in ADL was investigated by linear regression models (forced entry) containing each ADL measure as dependent variables. We also explored the pattern of association between cognitive performance and depressive symptoms through Pearson correlations to evaluate if the contribution of cognitive abilities and symptoms of depression to ADL performance is independent.

We used multiple linear regressions with a forced entry model to test whether the performance on specific cognitive domains and the intensity of depressive symptoms could predict the scores of the GADL components. *Z*-scores of executive functions, episodic memory, language/semantic memory, and visuospatial abilities, along with depressive symptoms (GDS-15 total score), were entered as independent variables in the models. In addition, we carried out ordinal regression analysis to assess whether the cognitive factors and depressive level predict the performance on each specific item of the GADL. Effect sizes were estimated by the adjusted *R*^2^ (linear regression) or Nagelkerke Pseudo-*R*^2^ (ordinal regression).

## Results

**Table 3** shows the sociodemographic, clinical data, ADL, and *Z*-scores for individual cognitive domains, according to diagnosis. Group comparisons indicate no differences in sociodemographic measures (*p* > 0.05), but significant differences in cognitive (*p* < 0.01), and ADL (*p* < 0.05) measures. The normal aging group outperformed both MCI and AD groups in cognitive measures, except for language/semantic memory compared with MCI patients. MCI patients showed higher scores than AD patients in all cognitive measures. Differences in ADL occurred only between AD and the other groups.

**Table 3:** Participants’ description and group comparisons.

Measures	NA (*N* = 96)	MCI (*N* = 85)	AD (*N* = 93)	*F*/χ^2^	Comparisons^2^
	*M* (SD)	*M* (SD)	*M* (SD)		
Age	72.61 (7.76)	73.18 (8.46)	73.18 (8.46)	1.80	-
Education	5.22 (4.29)	4.71 (4.00)	4.82 (3.46)	0.48	-
GDS-15	4.33 (3.95)	2.94 (2.84)	3.82 (3.22)	3.81	-
Sex (% Female)	67%	60%	55%	2.59^1^	-
MMSE	25.75 (3.85)	23.52 (3.62)	20.59 (3.98)	42.58^∗∗^	NA > MCI > AD
Language/Semantic memory	-0.34 (1.20)	-0.76 (0.96)	-1.69 (1.16)	27.28^∗∗^	NA = MCI > AD
Episodic memory	-0.28 (0.80)	-1.22 (0.72)	-1.76 (0.65)	93.47^∗∗^	NA > MCI > AD
Visuospatial abilities	-0.31 (1.08)	-0.84 (1.04)	-1.47 (1.05)	30.38^∗∗^	NA > MCI > AD
Executive functions	-0.58 (1.34)	-1.21 (1.05)	-2.25 (1.09)	46.91^∗∗^	NA > MCI > AD
GADL self-care	9.94 (0.32)	9.99 (0.11)	9.78 (0.87)	4.01^∗^	NA = MCI > AD
GADL domestic	7.68 (0.86)	7.41 (1.20)	5.74 (2.19)	19.61^∗∗^	NA = MCI > AD
GADL complex	7.55 (1.27)	6.91 (1.47)	4.35 (2.57)	59.61^∗∗^	NA = MCI > AD
GADL global score	25.16 (2.06)	24.03 (2.34)	19.88 (3.92)	75.03^∗∗^	NA = MCI > AD

*0.05, **<0.001. NA, Normal aging; MCI, Mild cognitive impairment; AD, Alzheimer’s disease; M, Mean; SD, Standard-deviation; GDS-15, Geriatric Depression Scale 15 items; GADL, General Activities of Daily Living Scale; ^1^Chi-Square test; ^2^One-Way ANOVA and Sidak’s post hoc test (p < 0.05).

**FIGURE 1:**
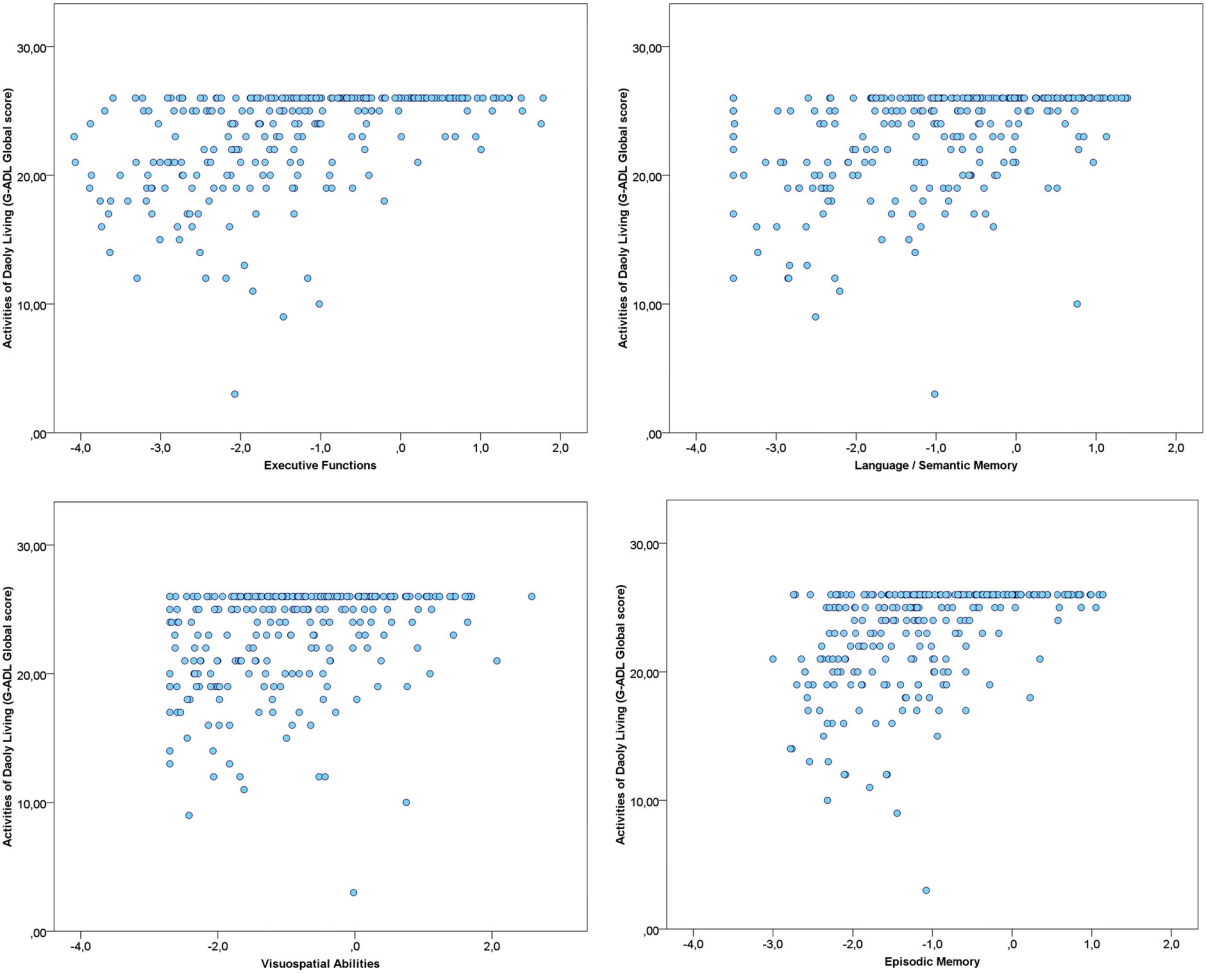
**Association between Cognitive Factors and General Activities of Daily Living Scale (GADL).** The correlations of cognitive factors with functional performance were all significant (*p* < 0.001). Executive functions showed the strongest correlation with GADL total score (*r* = 0.478), followed by episodic memory (*r* = 0.449), language/semantic memory (*r* = 0.410), and visuospatial abilities (*r* = 0.302). The number of dots in the scatterplot differs from the sample size due to superposed values.

**FIGURE 2:**
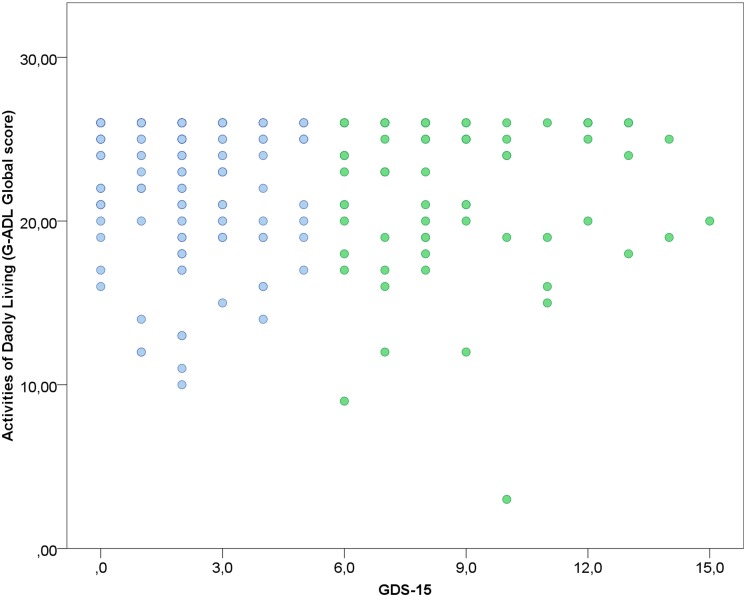
**Association between depression and GADL scores.** A weak correlation was observed between depressive status and GADL total score (*r* = -0.151, *p* = 0.013). In the scatterplot, blue dots represent the participants under the cut-off for depression according to the Geriatric Depression Scale and green dots the participants above the cut-off for depression. The number of dots in the scatterplot differs from the sample size due to superposed values.

Correlations between each cognitive factor, depressive symptoms and the global score of the GADL are show in **Figures 1** and **2**. All correlations between cognitive measures and ADL were significant (*p* < 0.001) and moderate, but we found only a weak correlation between depressive symptoms and the functional measure (*r* = -0.151, *p* = 0.013). The correlations between depressive symptoms and cognitive performance were significant for executive functions (*r* = -0.192, *p* = 0.001), but we found no association with episodic memory (*r* = -0.019, *p* = 0.753), language/semantic memory (*r* = -0.085, *p* = 0.162) and visuospatial abilities (*r* = -0.039, *p* = 0.525). The influence of sociodemographic factors (age, education and gender) on ADL performance was not significant: GADL Complex (*F* = 1.31, *p* = 0.272), GADL Domestic (*F* = 1.13, *p* = 0.336), GADL Self-care (*F* = 1.56, *p* = 0.200), and the global score (*F* = 2.40, *p* = 0.068).

**Table 4** shows the predictors of GADL subscales scores. For the global score of the GADL the model was significant (*F* = 22.90, *p* < 0.001, *R*^2^ = 0.29) and contained as predictors executive functions (*p* < 0.001), episodic memory (*p* < 0.001), and language/semantic memory (*p* = 0.011). The Self-Care model was not significant (*F* = 1.37, *p* = 0.234, *R*^2^ < 0.01). The model for Instrumental-Complex ADL was significant (*F* = 24.66, *p* < 0.001, *R*^2^ = 0.30) and contained as predictors executive functions (*p* = 0.007), episodic memory (*p* < 0.001), and language/semantic memory (*p* = 0.001). The model for Instrumental-Domestic was also significant (*F* = 14.39, *p* < 0.001, *R*^2^ = 0.19) and involved executive functions (*p* < 0.001) and episodic memory (*p*-0.009) as significant predictors.

**Table 4:** Linear regression models of cognitive function and depressive symptoms as predictors of different ADL.

*F*	df	*p*	*R*^2^	Predictors	Standard β	*p*
**GADL: Global score**						
22.90	(5,268)	<0.001	29%	Executive functions	0.30	<0.001
				Episodic memory	0.25	<0.001
				Language/Semantic memory	0.18	0.011
				Visuospatial abilities	-0.13	0.084
				Depressive Symptoms	-0.08	0.140
**GADL: Self-care score**						
1.37	(5,268)	0.234	<1%	Executive functions	0.20	0.039
				Episodic memory	-0.02	0.780
				Language/Semantic memory	-0.01	0.931
				Visuospatial abilities	-0.14	0.116
				Depressive symptoms	-0.05	0.299
**GADL: Instrumental-domestic score**						
14.39	(5,268)	<0.001	19%	Executive functions	0.33	<0.001
				Episodic memory	0.18	0.009
				Language/Semantic memory	0.09	0.211
				Visuospatial abilities	-0.10	0.237
				Depressive symptoms	-0.05	0.402
**GADL: Instrumental-complex score**						
24.66	(5,268)	<0.001	30%	Executive functions	0.22	0.007
				Episodic memory	0.28	<0.001
				Language/Semantic memory	0.23	0.001
				Visuospatial abilities	-0.12	0.120
				Depressive symptoms	-0.08	0.108

*ADL, Activities of daily living; GADL, General Activities of Daily Living Scale; df, Degrees of freedom.*

**Tables 5** and **6** show the role of specific cognitive factors and depressive symptoms as predictors of specific IADL. Since the cognitive factors and depressive symptoms were unrelated to GADL Self-Care scores, the analyses were carried out for Instrumental-Domestic and Instrumental-Complex activities only.

**Table 5:** Ordinal regression analysis of cognitive functions and depressive symptoms as predictors of Instrumental-Domestic activities of daily living.

χ^2^	df	*p*	*R*^2^	Predictors	Est.	SE	*p*
**Do simple domestic chores**							
48.70	5	<0.001	26%	Executive functions	-0.74	0.22	0.001
				Episodic memory	-0.74	0.28	0.008
				Language/Semantic memory	-0.24	0.19	0.207
				Visuospatial abilities	0.49	0.25	0.065
				Depressive symptoms	0.06	0.05	0.224
**Do personal laundry**							
45.91	5	<0.001	24%	Executive functions	-0.72	0.20	<0.001
				Episodic memory	-0.61	0.25	0.015
				Language/Semantic memory	-0.08	0.18	0.649
				Visuospatial abilities	0.29	0.23	0.209
				Depressive symptoms	0.11	0.05	0.025
**Use the telephone**							
52.97	5	<0.001	28%	Executive functions	-0.57	0.20	0.005
				Episodic memory	-0.37	0.26	0.147
				Language/Semantic memory	-0.45	0.18	0.014
				Visuospatial abilities	0.14	0.24	0.558
				Depressive symptoms	0.15	0.05	0.003
**Prepare meals**							
42.53	5	<0.001	22%	Executive functions	-0.64	0.19	0.001
				Episodic memory	-0.47	0.24	0.046
				Language/Semantic memory	-0.01	0.17	0.978
				Visuospatial abilities	0.01	0.22	0.950
				Depressive symptoms	0.02	0.05	0.668

*χ^2^, Chi-Square test; df, Degrees of freedom; R^2^, Nagelkerke pseudo R-Square; Est., Ordinal logistic regression model estimate; SE, Standard error.*

**Table 6:** Ordinal regression analysis of cognitive functions and depressive symptoms as predictors of Instrumental-Complex activities of daily living.

χ^2^	df	*p*	*R*^2^	Predictors	Est.	SE	*p*
**Manage finances**							
67.43	5	<0.001	30%	Executive functions	-0.30	0.18	0.089
				Episodic memory	-0.81	0.23	<0.001
				Language/Semantic memory	-0.43	0.16	0.008
				Visuospatial abilities	0.05	0.20	0.801
				Depressive symptoms	0.11	0.05	0.016
**Shopping**							
91.15	5	<0.001	38%	Executive functions	-0.42	0.18	0.018
				Episodic memory	-0.95	0.24	<0.001
				Language/Semantic memory	-0.60	0.16	<0.001
				Visuospatial abilities	0.31	0.21	0.139
				Depressive symptoms	0.10	0.05	0.030
**Use of medication**							
96.07	5	<0.001	38%	Executive functions	-0.71	0.18	<0.001
				Episodic memory	-0.99	0.22	<0.001
				Language/Semantic memory	-0.27	0.15	0.077
				Visuospatial abilities	0.36	0.20	0.065
				Depressive symptoms	0.06	0.04	0.162
**Go out alone and use transports**							
75.93	5	<0.001	33%	Executive functions	-0.51	0.18	0.004
				Episodic memory	-0.72	0.22	0.001
				Language/Semantic memory	0.23	0.20	0.252
				Visuospatial abilities	-0.40	0.16	0.012
				Depressive symptoms	0.14	0.04	0.001

*χ^2^, Chi-Square test; df, Degrees of freedom; R^2^, Nagelkerke pseudo R-Square; Est., Ordinal logistic regression model estimate; SE, Standard error.*

All Instrumental-Domestic activities were associated with executive functions (*p* < 0.05) and, except for the independence in doing personal laundry, with episodic memory (*p* < 0.05). Language/semantic memory was related only to the correct use of the telephone (*p* = 0.014). Visuospatial abilities were not significantly related to any Domestic ADL (all *p* > 0.05). Depressive symptoms were predictors of doing personal laundry (*p* = 0.025) and difficulties using the telephone (*p* = 0.003). The effect sizes for the comparisons were moderate-large, ranging from 22 to 28% of explained variance.

Episodic memory was a significant predictor of all Instrumental-Complex ADL (*p* < 0.05). Executive Functions followed a similar pattern (*p* < 0.05), but was not predictive of the individual’s ability to manage finances (*p* = 0.089). Language/Semantic Memory was a significant predictor of independence in performing simple shopping (*p* < 0.001) and managing finances (*p* = 0.008). The Visuospatial abilities were a significant predictor of the ability to go out alone and use transportation (*p* = 0.012). Depressive symptoms predicted the ability to shop (*p* = 0.030), handle financial matters (*p* = 0.016), and go out alone using transportation (*p* < 0.001), but not the medication management (*p* = 0.162). The effect sizes of these models were large, ranging from 30 to 38% of explained variance.

## Discussion

In the present study, we showed distinct cognitive domains having a significant impact on ADL in older adults with a wide range of cognitive deficits. Executive functioning and Episodic Memory showed the strongest significant association with functional performance. Language/Semantic Memory contributed to complex aspects of ADL and visuospatial abilities contributed only to a specific instrumental activity. Depressive symptoms had a significant influence on more complex ADL such as handling finances. Self-care ADL were not related to cognitive performance. Executive functions showed only a weak correlation with depressive symptoms. The results are in agreement with previous studies and highlight the close relationship between deficits in specific cognitive domains and functional loss.

Episodic memory and executive functions were the most important predictors of domestic ADL performance. The execution of these activities requires skills related to the identification and ordering of different steps necessary to achieve the final goal (e.g., different steps to prepare a meal) or recalling information after a period of time or in face of distractors (e.g., remembering what of house cleaning was already done and what was not). These behaviors are intrinsically related to different aspects of executive functioning and episodic memory.

Similar to our findings, previous studies showed executive functions and episodic memory tests as significant predictors of the ability to cook and to do household chores (Farias et al., 2003; Matsuda and Saito, 2005; Mariani et al., 2008). Jefferson et al. (2006) found association between verbal fluency and food preparing, cognitive flexibility, and selective attention with doing laundry, but no correlations between executive functions and house cleaning. An interesting research using extrapyramidal signs and structural brain imaging found that, controlling these previous factors and sociodemographic aspects, the performance in tests of memory and executive functions was still associated with cooking, and specific measures of executive functions with the ability to perform simple domestic chores (Bennett et al., 2006). We found an association between depressive symptoms with doing laundry. We hypothesize that this might be a sample bias. Most of the older adults assessed in our study had a very low socioeconomic level and usually do not have laundry machines. Since they do the laundry manually, the association with depressive symptoms may be due to lack of energy or apathy since this activity is very physically demanding.

We found significant associations between executive functions, language/semantic memory, depressive symptoms, and telephone use. The engagement of these specific cognitive functions may reflect the necessity of communication to perform this activity. The neuropsychological battery used in this study included instruments related to expressive language, comprehension, and access to semantic and phonological lexicons. Therefore, we expected that ADL related to communicative skills would be influenced by the performance on these cognitive domains (Taler and Phillips, 2008; Razani et al., 2011). Farias et al. (2003), however, found motor praxis as the only predictor of telephone use. Depressive symptoms also influenced telephone use in our study. Social isolation, a common characteristic of elderly persons with depression (Corcoran et al., 2013), may reduce the individual willingness to actively pursue contact with other people, leading to impairments in this specific activity.

Two of the Complex-ADL activities involve management of finances. Episodic memory and language/semantic memory predicted financial management, while executive functions, episodic memory, and language/semantic memory predicted shopping ability. These are complex activities and involve several cognitive processes (Marson et al., 2009). Sherod et al. (2009) decomposed financial managing it in basic financial skills, financial conceptual knowledge, financial transactions, checkbook control, banking control, and financial judgment. The authors identified the cognitive predictors of financial capacity in the spectrum of normal aging, MCI, and AD using a specific questionnaire for financial management and a comprehensive battery of neuropsychological tests. Their findings suggest that arithmetic skills (which relies on working memory) are the main predictor of financial capacity. Jefferson et al. (2006) found an association between selective attention and the ability to shop and to control finances. Tasks related to episodic memory, basic math skills, and a test related to language/semantic memory predicted financial control in Matsuda and Saito (2005) study. Razani et al. (2011) evaluated skills related to the management of money and found similar predictors to the present study: how to write out checks was associated with language, control the checkbook was related to executive functions, and shopping ability was associated with memory and executive functions. Motor praxis also might be related to financial management (Farias et al., 2003). Additionally, we observed depressive symptoms predicting worse performance in the management of finances. This finding is in contrast to Farias et al. (2003) who found no significant association between depressive symptoms and management of finances.

The correct use of medications was associated with executive functions and episodic memory in the present research in accordance with previous studies (Maddigan et al., 2003; Sino et al., 2014). A very common complaint by patients with memory impairment is to forget when to take medications or difficult to remember if he/she has already take it or not. This emphasizes the importance of different aspects of the episodic memory for the correct maintenance of medical care routine (Matsuda and Saito, 2005). Complex medication routines may demand more executive control (Maddigan et al., 2003). A study reported that performance in executive functions tests (including working memory) was a significant predictor of medication use in older adults (Insel et al., 2006). However, Jefferson et al. (2006) did not find any significant associations in this direction. Compensatory strategies might explain these discrepancies. Carlson et al. (2005) tested two objective measures of medication use capacity (schedule and pillbox) and found a significant association between memory performance and the schedule strategy and between executive functions and the pillbox.

We found executive functions, episodic memory, visuospatial skills, and depressive symptoms as predictors of ADL related to going out of home alone to distant locations using transportation. Different aspects of executive functions such as planning, cognitive flexibility, and selective attention were associated with transportation in the study of Jeferson et al. (2006). Deficits in these functions were associated with impairment in “visually” dependent activities such as driving, orientation, and transport use (Silva et al., 2009; Farley et al., 2011). However, despite the expected relationship between visuospatial abilities and the ability to travel long distances, few studies have found a significant direct association between the neuropsychological performance and its functional counterpart. Our findings provide a model in which visuospatial and navigation abilities depend on executive functions, episodic memory, and visuospatial skills. Matsuda and Saito (2005) found similar results in the Japanese population. Depressive symptoms also predicted performance in the ability to travel. As in telephone use, social isolation might mediate the association between depressive symptoms and functional performance on this task.

In our view, the strengths of the current study are the relatively large sample size, the heterogeneity of the participants and the use of fine-grained cognitive and functional measures. The use of cognitive factors validated for this population instead of tests’ raw scores allow the construction of more precise conceptual models and is easier to be generalized for other settings, since the data is analyzed in the cognitive construct level and can be represented by different cognitive tests. However, the present results should be viewed in light of its limitations. Although the neuropsychological measures used comprise four specific cognitive domains, the protocol had no specific measure of processing speed, a cognitive domain related to functional performance and a potential mediator of depression influence on daily functioning (Brown et al., 2013). The executive functions factor adopted in this study may be related to processing speed in our population since processing speed and executive functions influence verbal fluency in an independent way in older adults with low formal education (de Paula et al., 2013b). Therefore, the relationship between the executive function domain and functional performance could be secondary to its processing speed component. Additional studies including specific measures of processing speed should evaluate its impact on ADL. Additionally, the present study has a cross-sectional design, which limits the interpretation of our findings. Future studies with a longitudinal design are necessary to assess the impact of changes in specific cognitive functions on the performance of specific ADL. Another aspect is the lack of an ecological functional measure since our work relies on scales of caregiver report, which may result in different results.

## Conclusion

We found executive function and episodic memory as the cognitive domains most frequently related to impairment in general constructs of ADL. Nonetheless, language, semantic memory, and visuospatial abilities may influence specific functional aspects of ADL. The development of better predictive models can aid to the development of tailored and personalized rehabilitation programs to improve functional performance of subjects with neurocognitive disorders.

## Conflict of Interest Statement

The authors declare that the research was conducted in the absence of any commercial or financial relationships that could be construed as a potential conflict of interest.
